# We should not abandon therapeutic cooling after cardiac arrest

**DOI:** 10.1186/cc13817

**Published:** 2014-04-04

**Authors:** Kees H Polderman, Joseph Varon

**Affiliations:** 1 Department of Critical Care Medicine, University of Pittsburgh Medical Center, 3550 Terrace Street, Scaife Hall/6th floor, Pittsburgh, PA 15261, USA; 2 The University of Texas Health Science Center at Houston, Houston, TX 77030, USA; 3 The University of Texas Medical Branch at Galveston, Galveston, TX 77550, USA; 4 University General Hospital, Houston, TX 77054, USA

## 

Therapeutic hypothermia (TH) has been used to treat post-hypoxic brain injury after cardiac arrest (CA) since the late 1950s. In 2002, two landmark prospective, randomized controlled trials (RCTs) confirmed the efficacy of TH for this indication [1,2]. An 11-center trial in Europe reported 16% absolute improvement in outcome in patients with witnessed ventricular tachycardia/ventricular fibrillation (VT/VF) arrest with use of TH [1], and a four-center Australian study found 23% improvement [2]. More than 40 non-randomized studies have reported improved outcomes with TH [3]. A 5,317-patient registry in The Netherlands noted a 6.6% drop in mortality with TH implementation [4]. A Scandinavian registry with 986 patients reported 61% survival in witnessed VT/VF arrest, 92% with good neurological outcome [5]. A meta-analysis concluded that six patients had to be treated to achieve one additional case of good outcome [6]. On these bases, professional societies began recommending the use of TH in selected patients with CA [7,8]. A Cochrane Review supported these guidelines and conclusions [9]. Further supporting evidence came from the field of neonatology, in which seven RCTs showed improved outcomes with TH in newborns with hypoxic injury [3].

However, this evidence has been challenged [10]. The largest RCT had no strict temperature management in controls, who had an average temperature of 37.8°C [1]. Other criticisms included a lack of prior power calculation and a low rate of enrollment [10]. The second RCT successfully maintained normothermia in controls, but random assignment was by day of the month rather than per patient [2]. The critics stimulated the conduct of another, larger RCT, enrolling patients with witnessed CA regardless of initial rhythm, with predefined subgroup analyses [11]. This study enrolled 939 patients, who were cooled to either 33°C or 36°C. The results were unequivocally negative.

These findings completely contradict those of all previous studies. Should we accept the results because the study was well designed and larger than previous trials?

First, some important issues need to be clarified. TH was already the standard of care in participating hospitals, and the default option for patients not enrolled in the trial was TH. Indeed, many centers had published CA outcome data that were far better than those of this study [5,12,13]. Hence, admitting physicians might subconsciously have selected patients with the potential to benefit from receiving ‘routine’ TH rather than have screened them for trial eligibility. The Methods section [11] suggests that all patients were evaluated, but this seems questionable: 1,431 patients were screened, and 939 were enrolled; that is an unusually high enrollment rate of 66%. The study took place in 36 intensive care units in just over 2 years, and this translates to 18 patients screened and 12 enrolled per center per year, or one patient per center per month. This number seems extremely low.

Other potential problems include a rapid rate of active re-warming, from 33°C to 36°C in 6 hours, faster than in all previous trials; this can negate the benefits of TH [14,15]. The temperature graph, Figure 1 in the article [11], shows wide error bars, potentially indicating large temperature swings that can be harmful [14,15]. Also, it appears that many ‘favorable’ factors such as bystander-witnessed arrest and ‘shockable’ rhythm were more prevalent in the 36°C group but that ‘unfavorable’ factors such as circulatory shock and absence of pupillary and corneal reflexes were more common in the 33°C group [11]. The differences are small but may be cumulative. There was a greater prevalence of spontaneous hypothermia (before start of active cooling) in the 33°C group, potentially indicating greater severity of brain injury with diminished shivering response [14,15]. There were more seizures in the 33°C group, in spite of the well-recognized anti-seizure effects of hypothermia [3]. More patients in the 33°C group met criteria for early withdrawal of care, again suggesting greater severity of injury [11].

The consequences of accepting these conclusions are momentous. We urge our colleagues not to abandon TH in favor of strict fever management (or, worse, no temperature management) on the basis of one study, until all relevant issues have been satisfactorily addressed.

### Abbreviations

CA: Cardiac arrest; RCT: Randomized controlled trial; TH: Therapeutic hypothermia; VT/VF: Ventricular tachycardia/ventricular fibrillation.

### Competing interests

The authors declare that they have no competing interests.

## References

[B1] Hypothermia after Cardiac Arrest Study Group Mild therapeutic hypothermia to improve the neurologic outcome after cardiac arrest N Engl J Med 2002 346 549 556 11856793 10.1056/NEJMoa012689

[B2] Bernard SA Gray TW Buist MD Jones BM Silvester W Gutteridge G Smith K Treatment of comatose survivors of out-of-hospital cardiac arrest with induced hypothermia N Engl J Med 2002 346 557 563 10.1056/NEJMoa003289 11856794

[B3] Polderman KH Induced hypothermia and fever control for prevention and treatment of neurological injuries Lancet 1955–1969 2008 371 10.1016/S0140-6736(08)60837-5 18539227

[B4] van der Wal G Brinkman S Bisschops LL Hoedemaekers CW van der Hoeven JG de Lange DW de Keizer NF Pickkers P Influence of mild therapeutic hypothermia after cardiac arrest on hospital mortality Crit Care Med 2011 39 84 88 10.1097/CCM.0b013e3181fd6aef 20959784

[B5] Nielsen N Hovdenes J Nilsson F Rubertsson S Stammet P Sunde K Valsson F Wanscher M Friberg H Hypothermia Network Outcome, timing and adverse events in therapeutic hypothermia after out-of-hospital cardiac arrest Acta Anaesthesiol Scand 2009 53 926 934 10.1111/j.1399-6576.2009.02021.x 19549271

[B6] Holzer M Bernard SA Hachimi-Idrissi S Roine RO Sterz F Müllner M Collaborative Group on Induced Hypothermia for Neuroprotection After Cardiac Arrest Hypothermia for neuroprotection after cardiac arrest: systematic review and individual patient data meta-analysis Crit Care Med 2005 33 414 418 10.1097/01.CCM.0000153410.87750.53 15699847

[B7] Peberdy MA Callaway CW Neumar RW Geocadin RG Zimmerman JL Donnino M Gabrielli A Silvers SM Zaritsky AL Merchant R Vanden Hoek TL Kronick SL American Heart Association Part 9: post-cardiac arrest care, American Heart Association Guidelines for Cardiopulmonary Resuscitation and Emergency Cardiovascular Care Circulation 2010 2010 S768 S786 20956225 10.1161/CIRCULATIONAHA.110.971002

[B8] Rittenberger JC Polderman KH Smith WS Weingart SD Emergency neurological life support: resuscitation following cardiac arrest Neurocrit Care 2012 17 S21 S28 10.1007/s12028-012-9750-9 22932988

[B9] Arrich J Holzer M Havel C Müllner M Herkner H Hypothermia for neuroprotection in adults after cardiopulmonary resuscitation Cochrane Database Syst Rev 2012 9 CD004128 10.1002/14651858.CD004128.pub3 22972067

[B10] Nielsen N Friberg H Gluud C Herlitz J Wetterslev J Hypothermia after cardiac arrest should be further evaluated — a systematic review of randomised trials with meta-analysis and trial sequential analysis Int J Cardiol 2011 151 333 341 10.1016/j.ijcard.2010.06.008 20591514

[B11] Nielsen N Wetterslev J Cronberg T Erlinge D Gasche Y Hassager C Horn J Hovdenes J Kjaergaard J Kuiper M Pellis T Stammet P Wanscher M Wise MP Åneman A Al-Subaie N Boesgaard S Bro-Jeppesen J Brunetti I Bugge JF Hingston CD Juffermans NP Koopmans M Køber L Langørgen J Lilja G Møller JE Rundgren M Rylander C Smid O Werer C Winkel P Friberg H TTM Trial Investigators Targeted temperature management at 33°C versus 36°C after cardiac arrest N Engl J Med 2013 369 2197 2206 10.1056/NEJMoa1310519 24237006

[B12] Larsson IM Wallin E Rubertsson S Cold saline infusion and ice packs alone are effective in inducing and maintaining therapeutic hypothermia after cardiac arrest Resuscitation 2010 81 15 19 10.1016/j.resuscitation.2009.09.012 19853352

[B13] Bro-Jeppesen J Kjaergaard J Wanscher M Pedersen F Holmvang L Lippert FK Møller JE Køber L Hassager C Emergency coronary angiography in comatose cardiac arrest patients: do real-life experiences support the guidelines? Eur Heart J Acute Cardiovasc Care 2012 1 291 301 24062920 10.1177/2048872612465588 PMC3760568

[B14] Polderman KH Mechanisms of action, physiological effects, and complications of hypothermia Crit Care Med 2009 37 S186 S202 10.1097/CCM.0b013e3181aa5241 19535947

[B15] Polderman KH Therapeutic hypothermia and controlled normothermia in the intensive care unit: practical considerations, side effects, and cooling methods Crit Care Med 2009 37 1101 1120 10.1097/CCM.0b013e3181962ad5 19237924

